# Supplementary material for: Evaluation of the Immunoprotective Capacity of Five Vaccine Candidate Proteins against Avian Necrotic Enteritis and Impact on the Caecal Microbiota of Vaccinated Birds
Source: Animals (Basel). 2023 Oct 26;13(21):3323. doi: 10.3390/ani13213323 (PMC10650611; doi:10.3390/ani13213323)
Supplement: Supplementary file 1 [file animals-13-03323-s001.zip › animals-2653677-supplementary.pdf]

Supporting Information

a

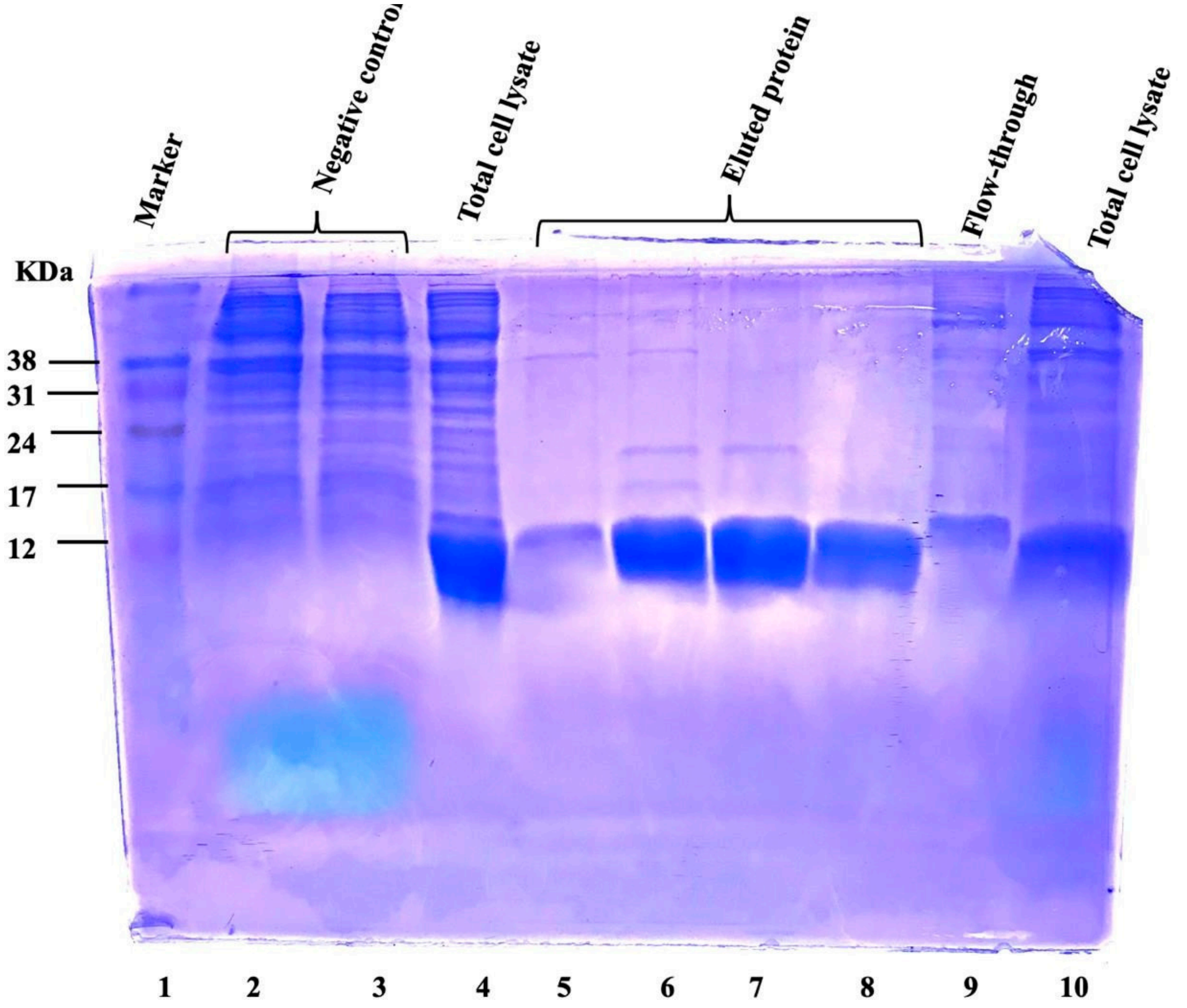

b

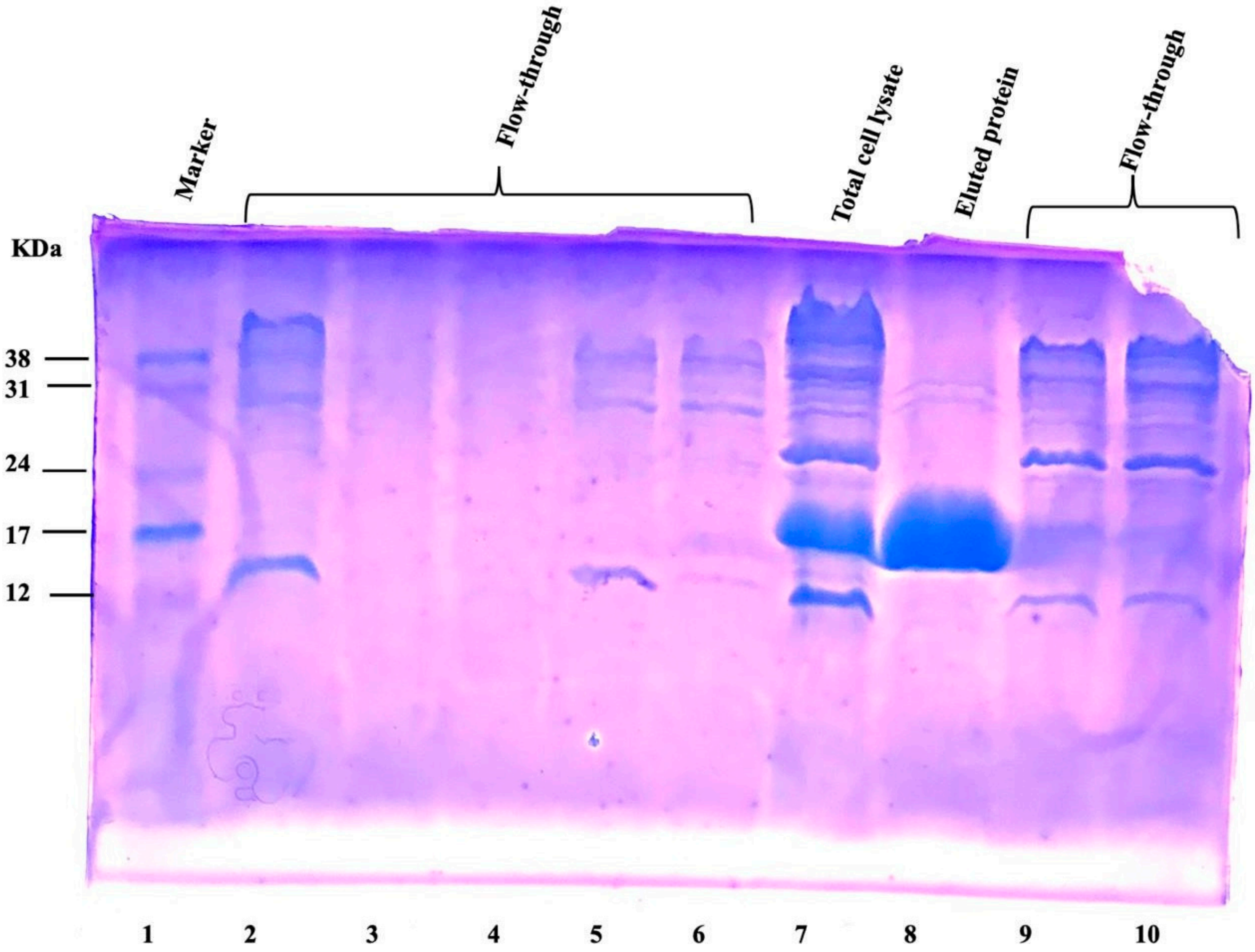

Marker
Flow-through
Total cell lysate
Eluted protein
Flow-through
KDa
38
31
24
17
12
1
2
3
4
5
6
7
8
9
10

c

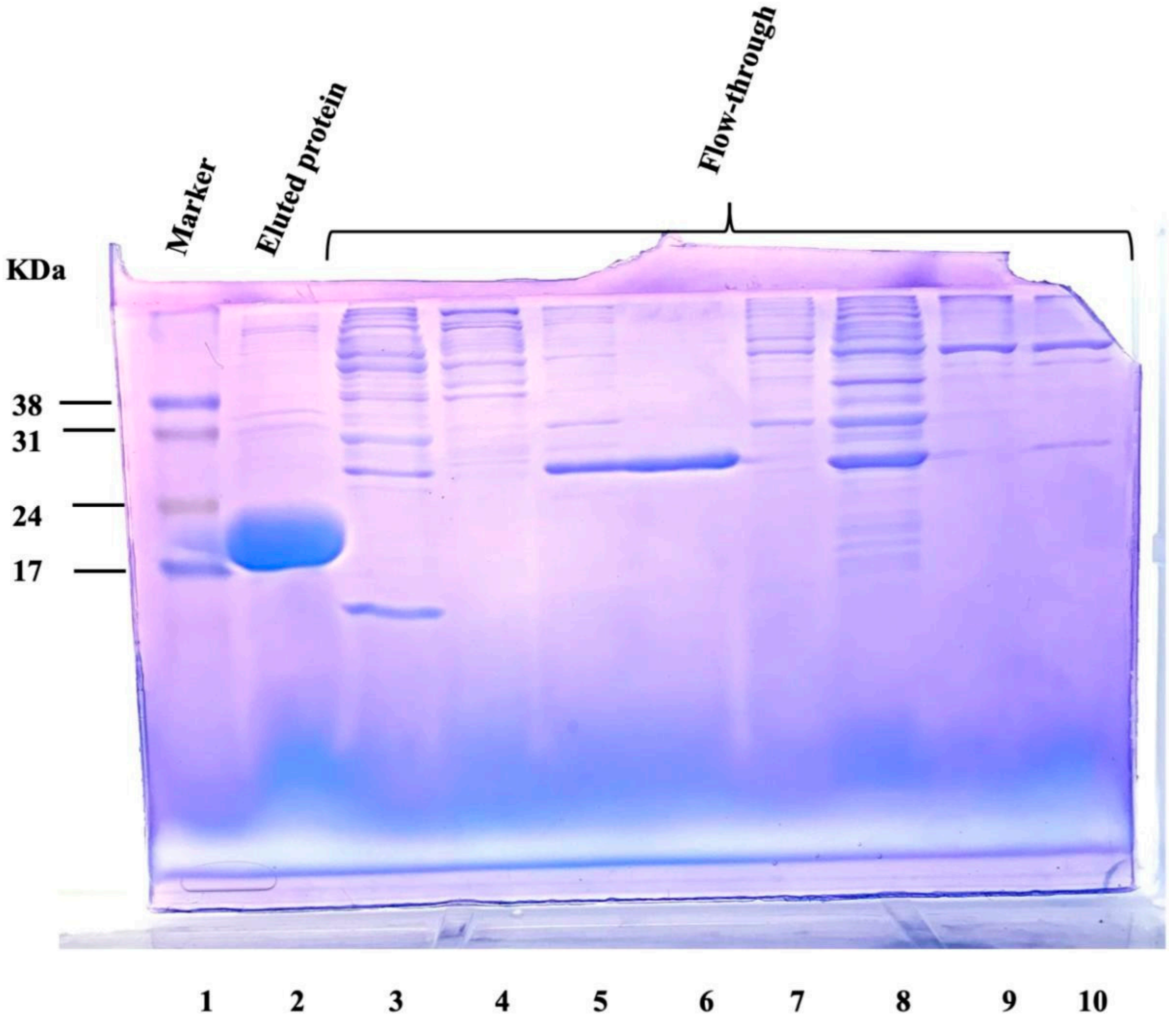
KDa
38
31
24
17
Marker
Eluted protein
Flow-through
1
2
3
4
5
6
7
8
9
10

d

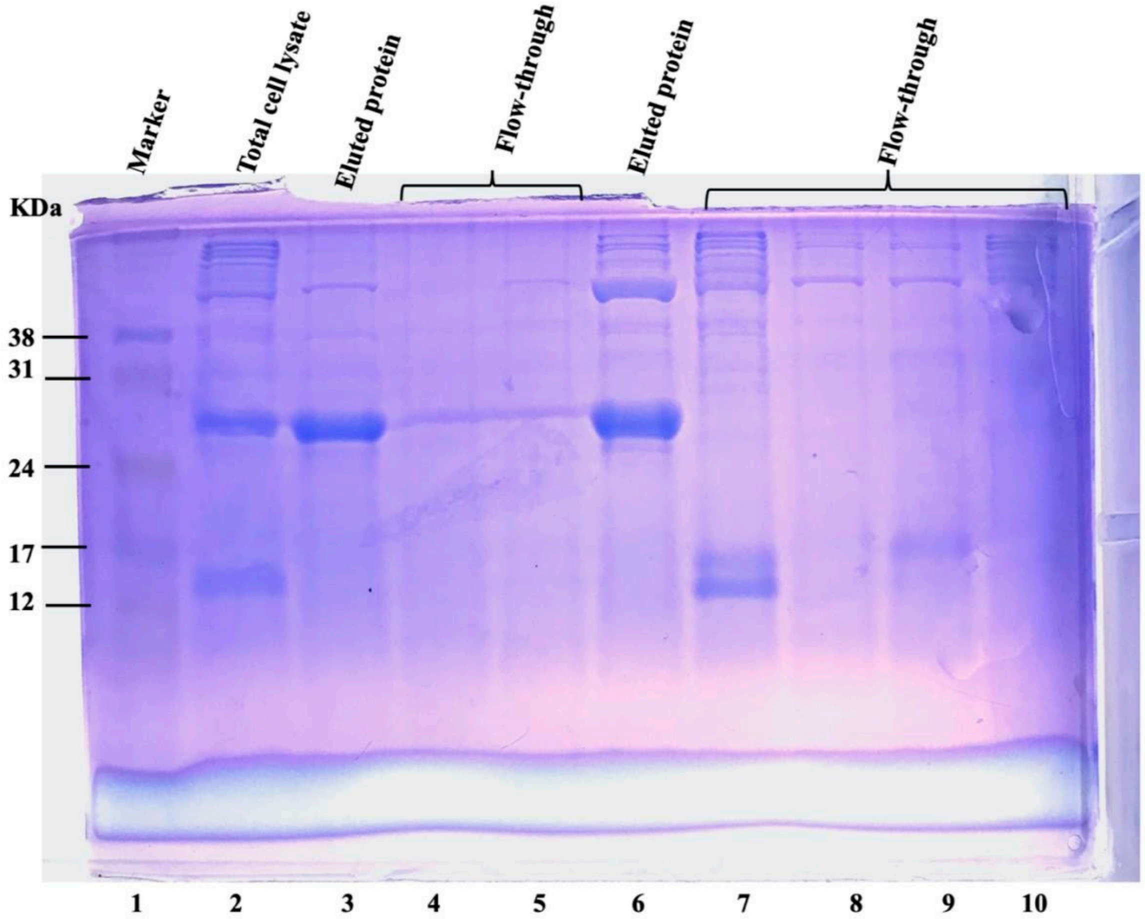

Marker
Total cell lysate
Eluted protein
Flow-through
Eluted protein
Flow-through
KDa
38
31
24
17
12
1
2
3
4
5
6
7
8
9
10

e

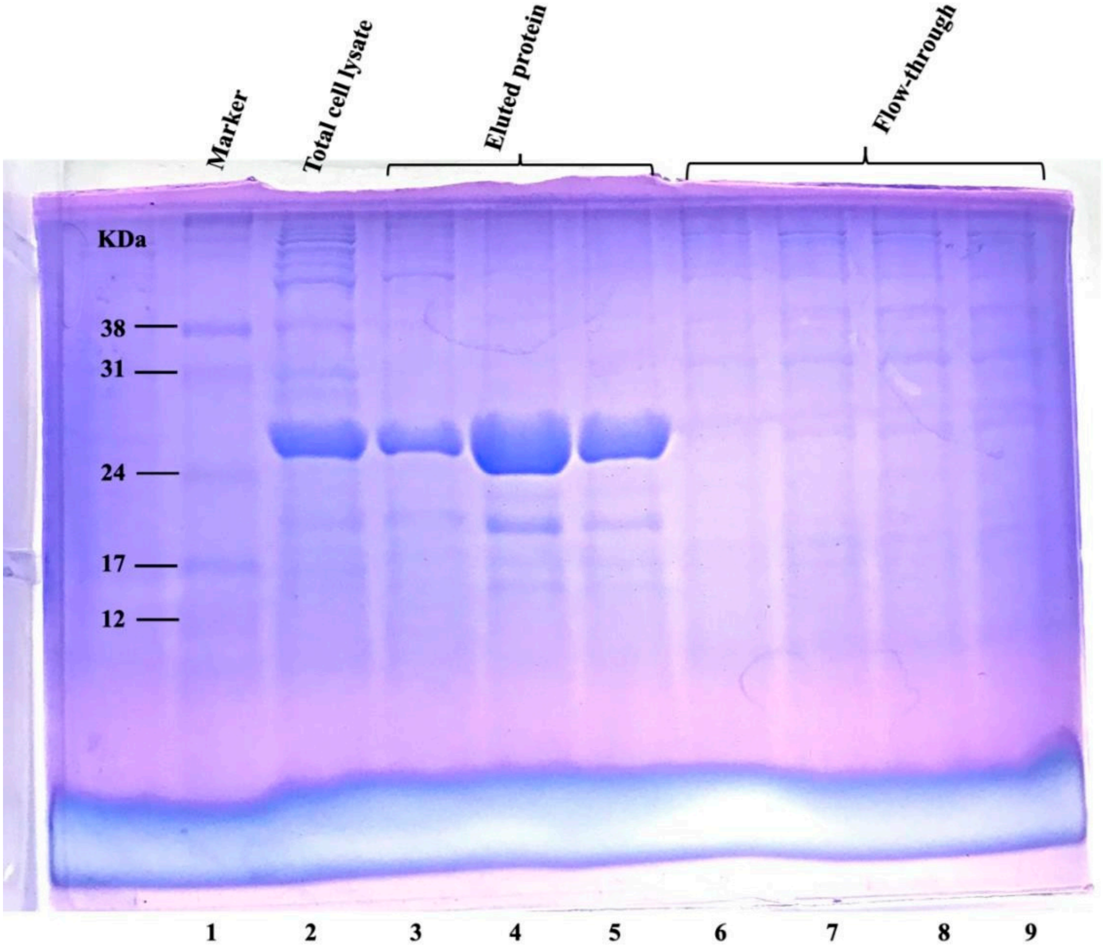

Figure S1. Coomassie blue stained SDS-PAGE gels showing purification of His-tagged recombinant proteins by Ni-NTA affinity chromatography. A volume of 15 µL of crude *E. coli* extract and fractions of (a) P153, (b) P264, (c) P509, (d) P537, and (e) P561 were loaded onto 15% SDS-polyacrylamide gels. The size of the protein of interest is 4 KDa higher than the predicted size due to the insertion of V5 and His-tag at the N-terminal of each coding sequence. Negative control sample in **Figure S1.a** corresponds to the crude cell lysate from BL21 cells that were transformed with pET empty vector.

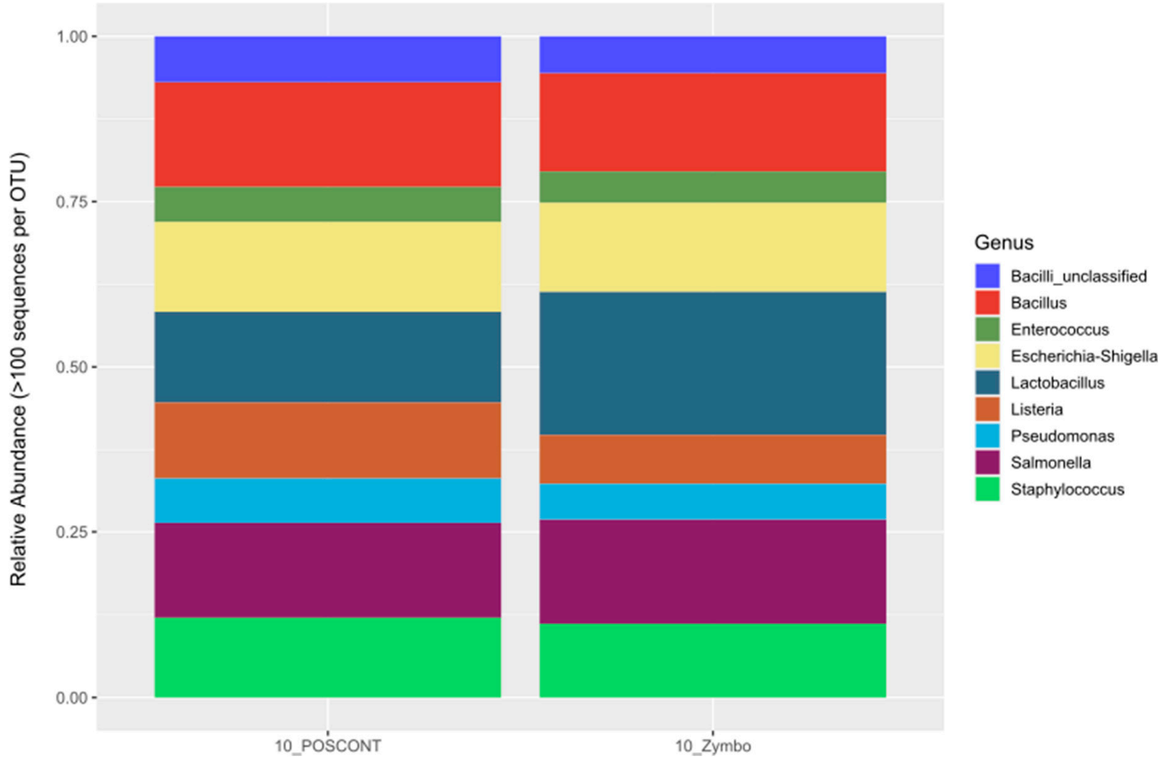

Figure S2. Stacked bar-plots showing the relative abundance of the major bacterial genera in positive controls.

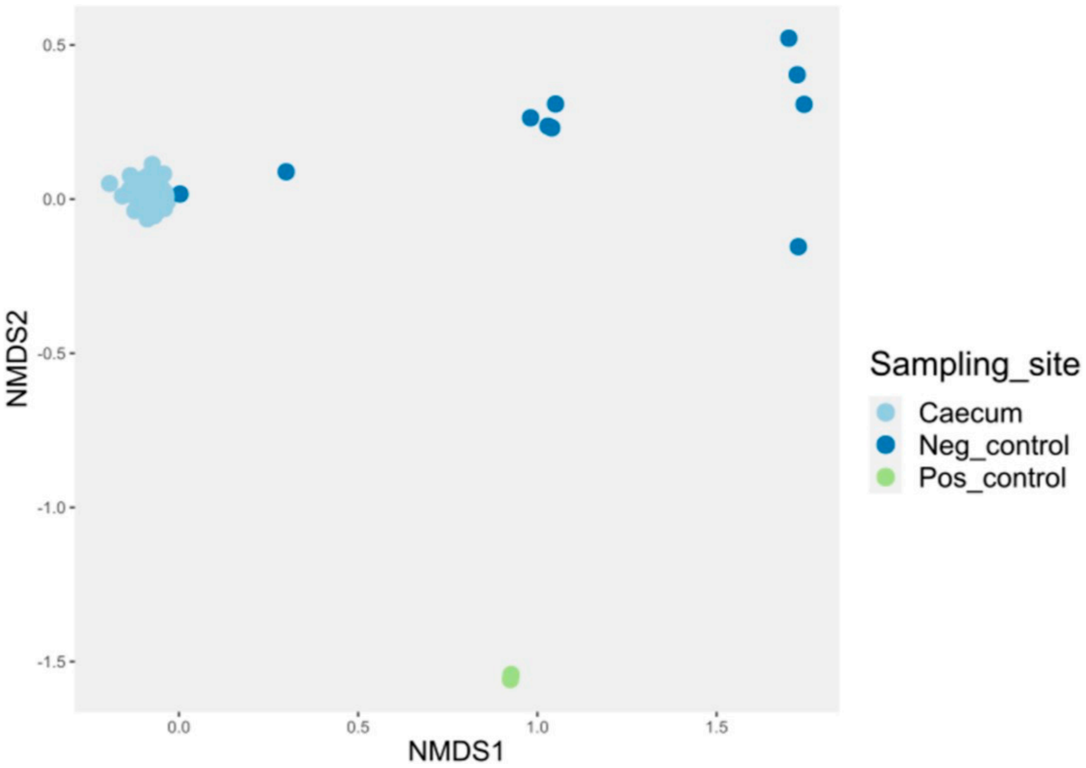

Figure S3. Non-metric multidimensional scaling plot (NMDS) showing the dissimilarity between cecum samples and controls using Bray–Curtis index.

A

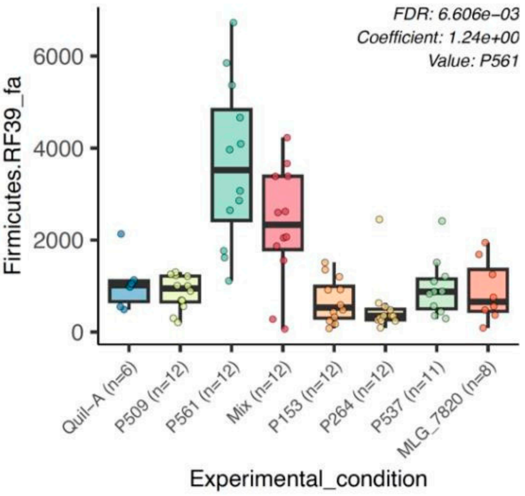
FDR: 6.606e−03
Coefficient: 1.24e+00
Value: P561
Firmicutes.RF39_fa
0
2000
4000
6000
Quil−A (n=6)
P509 (n=12)
P561 (n=12)
Mix (n=12)
P153 (n=12)
P264 (n=12)
P537 (n=11)
MLG_7820 (n=8)
Experimental_condition

B

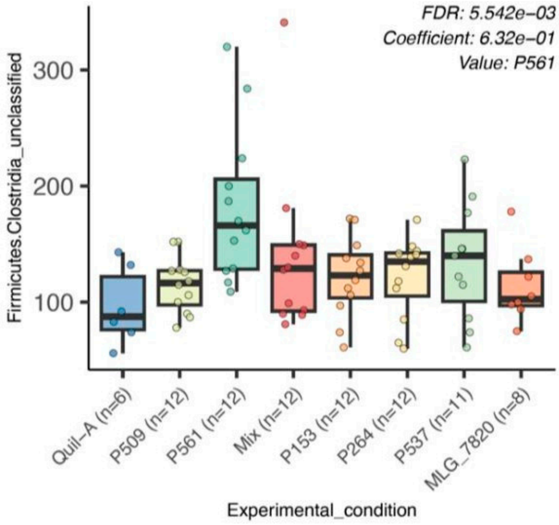
FDR: 5.542e−03
Coefficient: 6.32e−01
Value: P561
Firmicutes.Clostridia_unclassified
100
200
300
Quil−A (n=6)
P509 (n=12)
P561 (n=12)
Mix (n=12)
P153 (n=12)
P264 (n=12)
P537 (n=11)
MLG_7820 (n=8)
Experimental_condition

C

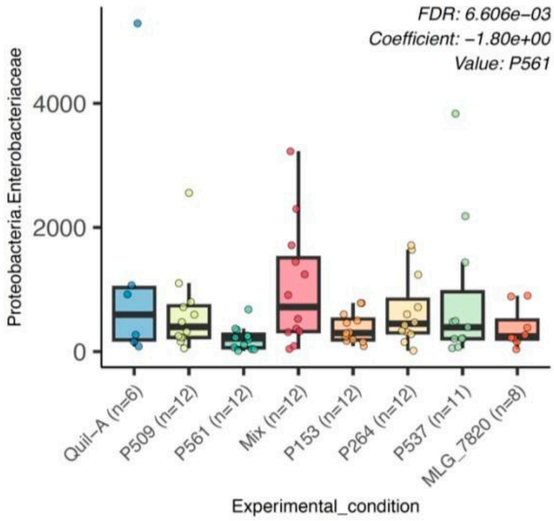
FDR: 6.606e−03
Coefficient: −1.80e+00
Value: P561
Proteobacteria.Enterobacteriaceae
0
2000
4000
Quil−A (n=6)
P509 (n=12)
P561 (n=12)
Mix (n=12)
P153 (n=12)
P264 (n=12)
P537 (n=11)
MLG_7820 (n=8)
Experimental_condition

D

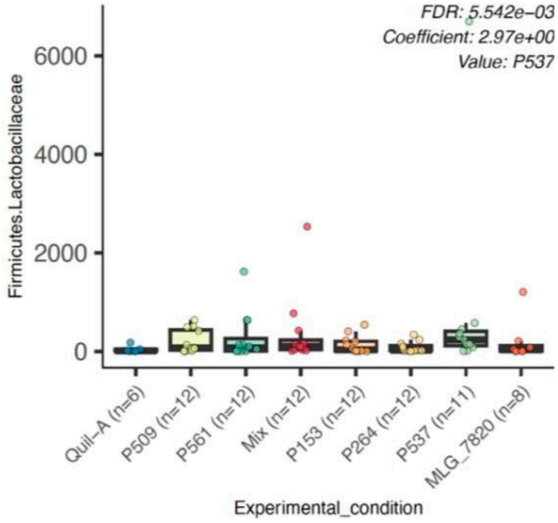
FDR: 5.542e−03
Coefficient: 2.97e+00
Value: P537
Firmicutes.Lactobacillaceae
0
2000
4000
6000
Quil−A (n=6)
P509 (n=12)
P561 (n=12)
Mix (n=12)
P153 (n=12)
P264 (n=12)
P537 (n=11)
MLG_7820 (n=8)
Experimental_condition

E

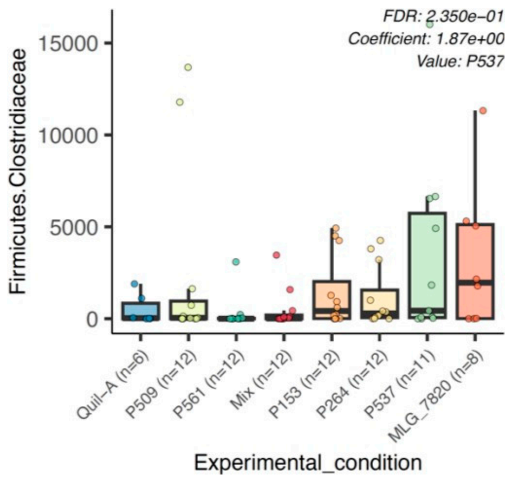

FDR: 2.350e−01
Coefficient: 1.87e+00
Value: P537
Firmicutes.Clostridiaceae
15000
10000
5000
0
Quil-A (n=6)
P509 (n=12)
P561 (n=12)
Mix (n=12)
P153 (n=12)
P264 (n=12)
P537 (n=11)
MLG_7820 (n=8)
Experimental_condition

F

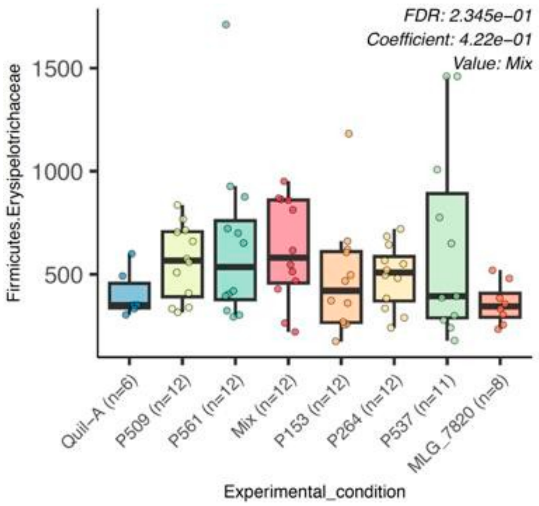

FDR: 2.345e−01
Coefficient: 4.22e−01
Value: Mix
Firmicutes.Erysipelotrichaceae
1500
1000
500
Quil-A (n=6)
P509 (n=12)
P561 (n=12)
Mix (n=12)
P153 (n=12)
P264 (n=12)
P537 (n=11)
MLG_7820 (n=8)
Experimental_condition

G

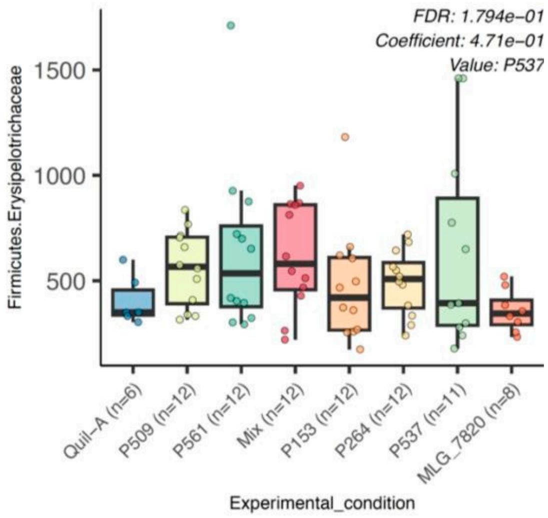

FDR: 1.794e−01
Coefficient: 4.71e−01
Value: P537
Firmicutes.Erysipelotrichaceae
1500
1000
500
Quil-A (n=6)
P509 (n=12)
P561 (n=12)
Mix (n=12)
P153 (n=12)
P264 (n=12)
P537 (n=11)
MLG_7820 (n=8)
Experimental_condition

H

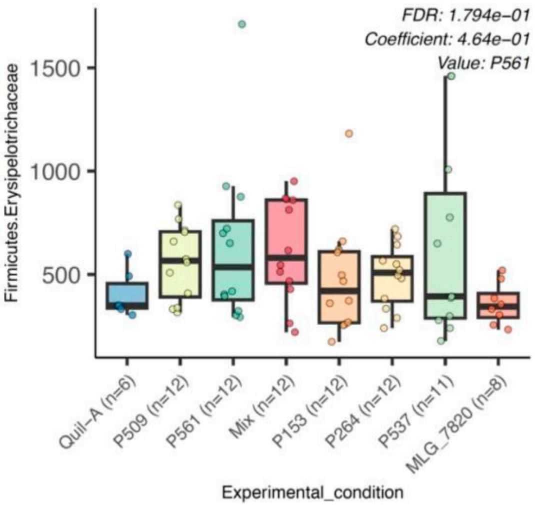

FDR: 1.794e−01
Coefficient: 4.64e−01
Value: P561
Firmicutes.Erysipelotrichaceae
1500
1000
500
Quil-A (n=6)
P509 (n=12)
P561 (n=12)
Mix (n=12)
P153 (n=12)
P264 (n=12)
P537 (n=11)
MLG_7820 (n=8)
Experimental_condition

I

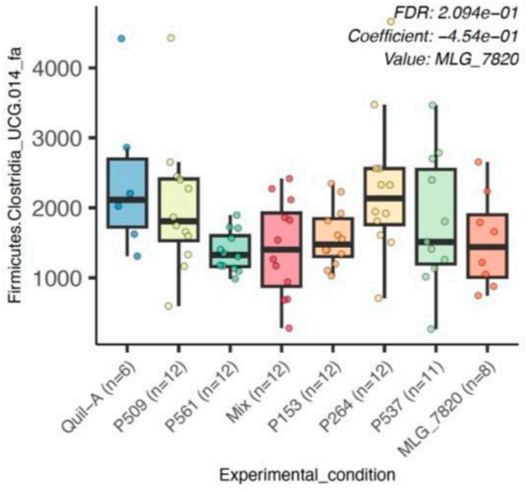

J

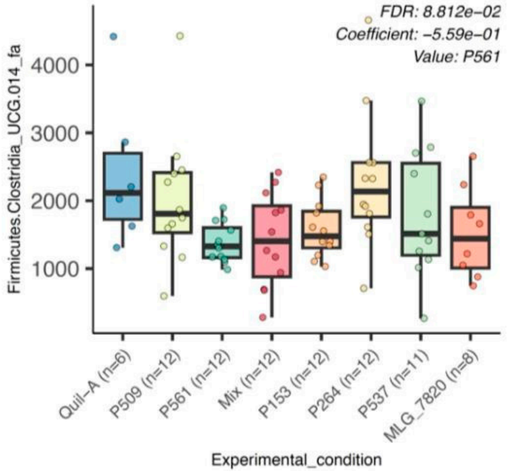

K

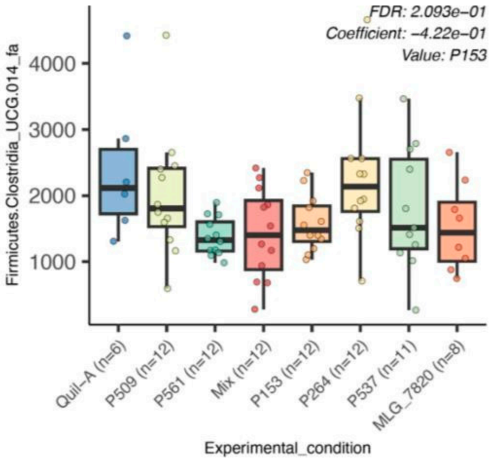

L

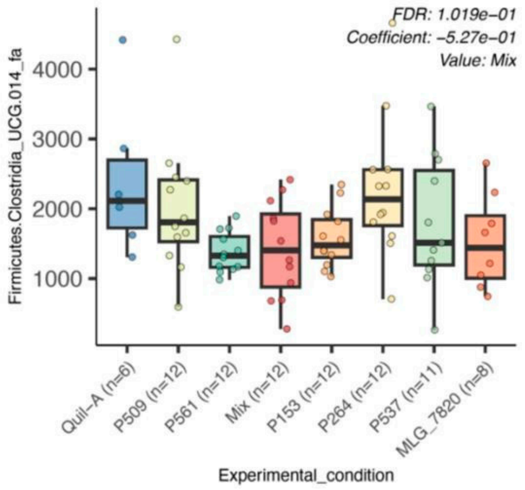

Figure S4. Statistically significant changes (p-value < 0.05) in key bacterial families of interest across experimental groups.

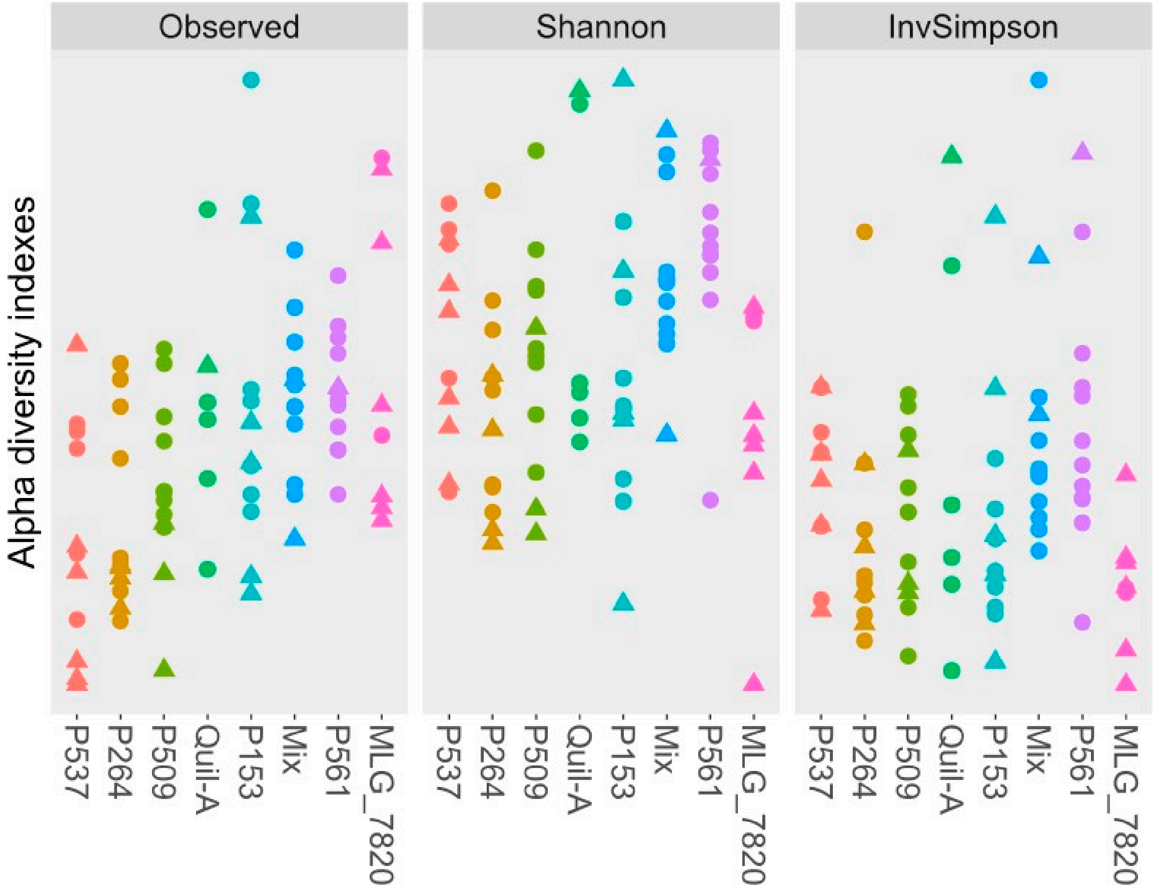

Figure S5. Alpha diversity measures among the samples at day 33, using Observed, Shannon, and Inverse Simpson indices.
